# Recent Development of Probiotic *Bifidobacteria* for Treating Human Diseases

**DOI:** 10.3389/fbioe.2021.770248

**Published:** 2021-12-22

**Authors:** Jun Chen, Xinyi Chen, Chun Loong Ho

**Affiliations:** Department of Biomedical Engineering, Southern University of Science and Technology (SUSTech), Shenzhen, China

**Keywords:** bifidobacteria, probiotic, therapeutic, genetic engineering, synthetic biology

## Abstract

*Bifidobacterium* is a non-spore-forming, Gram-positive, anaerobic probiotic actinobacterium and commonly found in the gut of infants and the uterine region of pregnant mothers. Like all probiotics, *Bifidobacteria* confer health benefits on the host when administered in adequate amounts, showing multifaceted probiotic effects. Examples include *B. bifidum, B. breve,* and *B. longum*, common *Bifidobacterium* strains employed to prevent and treat gastrointestinal disorders, including intestinal infections and cancers. Herein, we review the latest development in probiotic *Bifidobacteria* research, including studies on the therapeutic impact of *Bifidobacterial* species on human health and recent efforts in engineering *Bifidobacterium*. This review article would provide readers with a wholesome understanding of *Bifidobacteria* and its potentials to improve human health.

## Introduction

**FIGURE 1:**
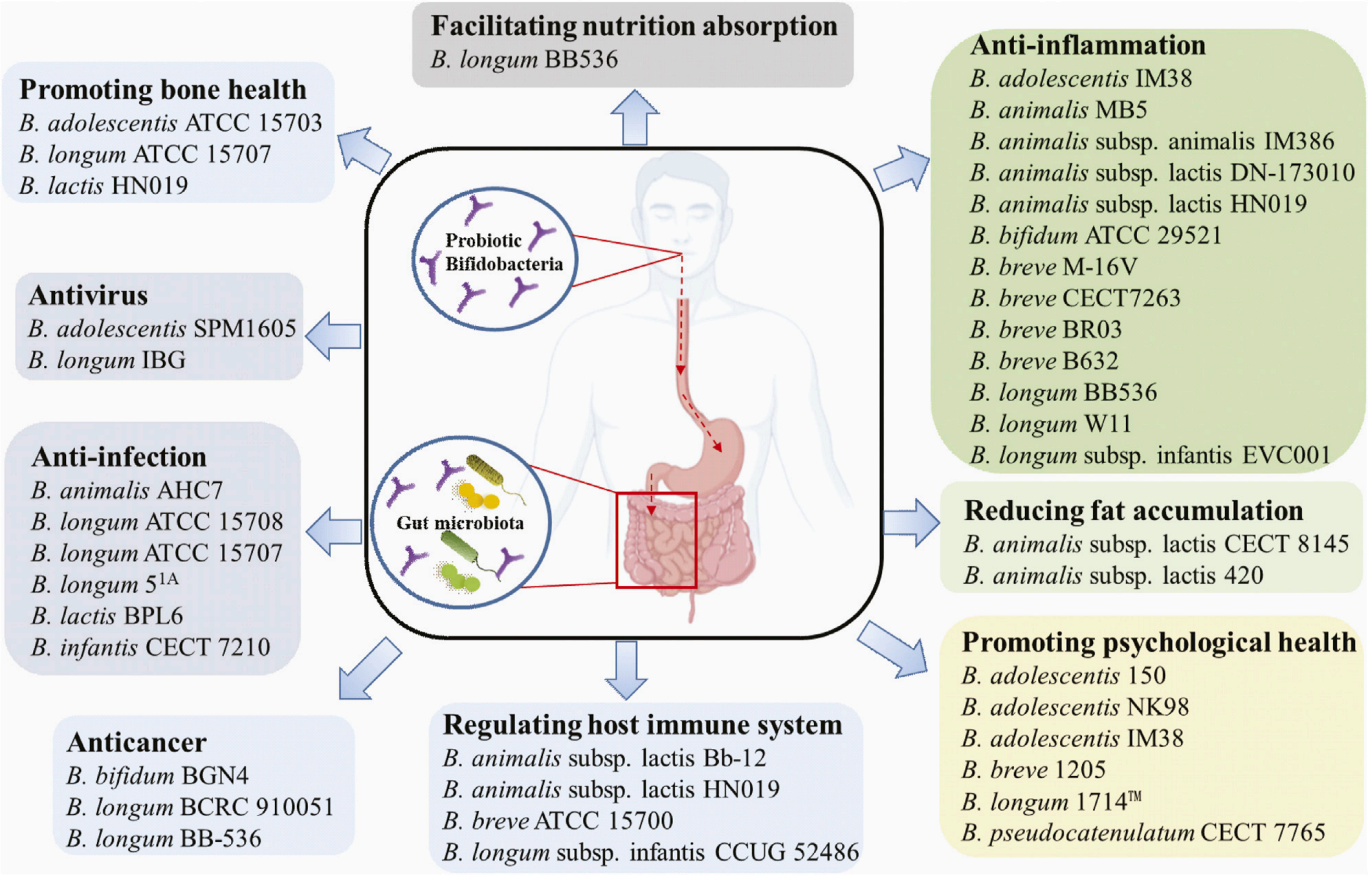
Beneficial effects of common probiotic Bifidobacterium strains.

Probiotic microorganisms are defined as living microorganisms that confer health benefiting properties to the host when administered adequately. Probiotics exert beneficial functions mainly through producing antimicrobial peptides, assimilating dietary fibers, regulating fat storage, modulating mucosal immunity, or regulating gut microbiota (Ku et al., 2016). For centuries, probiotics have been widely used in various functional foods, e.g., yoghurt, milk, cheese, infant formula, and dietary supplements. The most common probiotics include *Lactobacilli* and *Bifidobacteria*, which predominantly inhabit the animal or human intestinal tract (Hudault et al., 1994). *Bifidobacteria* are V- or Y-type branched, rod-shaped, immobile, non-spore-forming, Gram-positive, anaerobic, catalase-negative bacteria that belong to the family Bifidobacteriaceae and the phylum Actinobacteria. The *Bifidobacterium* genus currently includes over 90 species, excluding the unclassified species (Supplementary Table S1). *Bifidobacterium* was first isolated from breast-fed infant feces, but so far have been discovered from various ecological niches including sewage, fermented milk and anaerobic digestion facilities; nevertheless, the most frequent isolates are associated with the gastrointestinal tracts of humans and animals. The growth conditions (e.g., temperature, pH, oxygen level) of *Bifidobacteria* do not vary significantly among strains (Ruiz et al., 2011). For instance, the optimal growth temperature ranges between 36 and 38°C and 41–43°C for human- and animal-isolated strains, respectively. Additionally, the optimal growth pH is around pH 6.5–7.0, where *B. animalis* and *B. thermacidophilum* were found to be also metabolically active at pH 3.5–4.0. Most *Bifidobacterial* species are strict anaerobes, with a few exceptions, such as *B. boum*, *B. thermophilum*, *B. dentium* and *B. psychraerophilum* that tolerate microaerophilic environment. Hitherto, many probiotic *Bifidobacteria* have shown beneficial effects on humans or animals, e.g., antiinfection, anti-depression, regulating the host immune system, and facilitating host nutrition adsorption (Figure 1).

Furthermore, some probiotic *Bifidobacteria* are engineered to include other beneficial cellular functionalities and/or remove the disadvantageous properties. Herein, we provide a review of the various findings of *Bifidobacterium* probiotics’ therapeutic functions, metabolic pathways, and methods to engineer them. This review would provide readers with a better understanding of the beneficial functions of *Bifidobacterium* probiotics, including the genetically engineered strains.

### Beneficial Effects Exhibited by Probiotic *Bifidobacteria*

#### Antiinfection Activity

One prerequisite for becoming a probiotic strain is the capability of colonizing at a specific location, e.g., in the gastrointestinal tract, such that the probiotic strain can interact effectively with the host and the host microbiome. The colonization of probiotics outcompetes some pathogens and thus confers the host some protection against pathogenic infections. Various studies demonstrated that different species of *Bifidobacteria* exert antiinfection properties (Table 1). *B. longum* ATCC 15708 showed antimicrobial activity against many pathogens, including *Escherichia coli* O157:H7 ATTC 35150, *Salmonella typhimurium* ATTC 13311 and *Listeria monocytogenes* ATTC 19115 (Igbafe et al., 2020). *B. longum* BB536 protects against gut-derived sepsis caused by *Pseudomonas aeruginosa,* likely through interfering with the adherence of pathogens to intestinal epithelial cells (Matsumoto et al., 2008); BB536 ameliorates the upper respiratory infections in healthy pre-school children probably through modulating gut microbiota, i.e., increasing the abundance of the genus *Faecalibacterium* (Lau et al., 2018); also, another randomized, double-blind, placebo-controlled trial reveals that administration of BB536 in combination with the standard triple therapy (esomeprazole, amoxicillin, clarithromycin) improves the eradication rate of *Helicobacter pylori* infection in 63 patients (Chitapanarux et al., 2015). The administration of *B. lactis* BB-12 in early childhood reduces respiratory tract infections (Taipale et al., 2016). An administration of 5 billion colony-forming units of BB-12 twice a day for 1.5 years observed improved resistance to respiratory tract infections and low rates of developing fever throughout the infant’s growth period. *B. animalis* AHC7 was found to protect mice against *S. typhimurium* infection and prevent acute diarrhoea in dogs (Kelley et al., 2009; O’Mahony et al., 2009). The underlying mechanisms of the acute diarrhoea prevention of *B. animalis* AHC7 are due to the attenuation of proinflammatory transcription factor activation in response to infection (O'Mahony et al., 2010). *Bifidobacteria* are also used in displacing latent and chronic infectious strains. For instance, *B. longum* ATCC 15707 can prevent *Clostridium difficile*-infection (Yun et al., 2017), while *B. longum* 5^1A^ confers protection against *Klebsiella pneumoniae-*induced lung infection (Vieira et al., 2016). This protection conferred by *B. longum* 5^1A^ is due to the activation of the Toll-like receptor-signaling pathway, resulting in reactive oxygen species production. Similarly, *B. longum* 5^1A^ was found to reduce the Giardia-parasitic load in Mongolian gerbils (*Meriones unguiculatus*), making this strain a suitable prophylactic and therapeutic probiotic for promoting human and animal health (Fonseca et al., 2019).

**TABLE 1:** Beneficial effects displayed by common probiotic Bifidobacteria and the mechanisms involved.

Beneficial effects	Probiotic strains and the underlying possible mechanism	References
Antiinfection activity	*B. longum* ATCC 15708 may produce bacteriocins or bacteriocin-like compounds	Igbafe et al. (2020)
	*B. animalis* AHC7 may attenuate proinflammatory transcription factor activation in response to infection	O'Mahony et al. (2010)
	*B. longum* ATCC 15707 inhibits pathogen growth by decreasing pH values	Yun et al. (2017)
	*B. longum* 5^1A^ activates Toll-like receptor-signaling pathway and tunes the inflammatory response	Vieira et al. (2016)
	*B. longum* subsp. infantis CECT 7210 and *B. animalis* subsp. lactis BPL6 produce peptides with protease activity and modulate host immune response by increasing IL-10 and IgA	Moreno Muñoz et al. (2011), Gardini et al. (2016), Barba-Vidal et al. (2017)
Anti-virus activity	*B. adolescentis* SPM1605 inhibits the replication of Coxsackievirus B3	Kim et al. (2014)
	*B. longum* IBG may prevent viral adsorption	Botic et al. (2007); Colbère-Garapin et al. (2007); Lee et al. (2015)
Anticancer activity	*B. longum* BCRC 910051 enhances phagocytosis and proliferation of macrophages	Foo et al. (2011)
	The polysaccharide produced by *B. bifidum* BGN4 showed inhibitory effects on cancer cell lines	Ku et al. (2009)
	*B. longum* BB-536 may alter the physiological conditions in the colon, which further affects the metabolic activity of intestinal microflora	Reddy and Rivenson, (1993)
anti-inflammation	The colonized *B. breve* M-16V may regulate immune balance and inflammatory response	Wong et al. (2019)
	*B. adolescentis* IM38 inhibits NF-κB activation and lipopolysaccharide production	Lim and Kim, (2017)
	*B. animalis* MB5 can counteract neutrophil migration and partly reduce pathogen adhesion through regulating chemokine and cytokine expression	Roselli et al. (2006)
	*B. lactis* DN-173010 can decrease IL-1β level in gingival crevicular fluid	Kuru et al. (2017)
	*B. lactis* HN019 modulates the oral microbiota composition and reduces the magnitude of the inflammatory response	Oliveira et al. (2017), Ricoldi et al. (2017)
	*B. animalis* subsp. animalis IM386 assists in the digestion of lactose	Roškar et al. (2017)
	*B. bifidum* ATCC 29521 modulates NF-kB pathway and restores intestinal microbiome dysbiosis	Din et al. (2020)
	*B. breve* CECT7263 increases acetate and reduced trimethylamine production by gut microbiota	Robles Vera et al. (2020)
	*B. breve* BR03 and *B. breve* B632 decrease the production of pro-inflammatory cytokine TNF-α	Klemenak et al. (2015)
	*B. longum* BB536 inhibits the adherence of pathogens to intestinal epithelial cells	Matsumoto et al. (2008)
	*B. longum* W11 produces exopolysaccharides which increase the bacterial adhesion to the epithelium and increases intestinal motility	Di Pierro and Pane, (2021)
	*B. longum* infantis EVC001 prevents against enteric inflammation by decreasing proinflammatory cytokine release	Nguyen et al. (2021)
Promoting psychological health	*B. adolescentis* 150 produces the inhibitory neurotransmitter gamma-aminobutyric acid	Yunes et al. (2020), Dinan et al. (2013)
	*B. adolescentis* NK98 can regulate gut immune responses and microbiota composition	Jang et al. (2019)
	*B. adolescentis* IM38 can regulate the benzodiazepine site of the GABAA receptor or modulate stress-related cytokine	Jang et al. (2018)
	*B. breve* 1,205 probably induces metabolic changes via changing gut microbiota	Savignac et al. (2014) Allen et al. (2016)
	*B. longum* 1714™ modulates brain activity by regulating resting neural activity and neural responses	Allen et al. (2016), Savignac et al. (2014), Wang et al. (2019a)
	*B. pseudocatenulatum* CECT 7765 reduces nitric oxide release and regulates endocrine and immune mediators of the gut-brain axis	Moratalla et al. (2016), Mauricio et al. (2017), Agusti et al. (2018)
Reducing fat accumulation	*B. animalis* subsp. lactis CECT 8145 increases Akkermansia genus population in the gut	Martorell et al. (2016), Caimari et al. (2017), Pedret et al. (2019)
	*B. animalis* subsp. lactis 420 reduces translocation of gut microbes	Stenman et al. (2014)
Facilitating the host nutrition adsorption	*B. longum* BB536 alters the gut microbial community	Sugahara et al. (2015)
Promoting bone health	*B. longum* ATCC 15707 elevates the expression of *Sparc* and *Bmp*-2 genes	Parvaneh et al. (2015), Rodrigues et al. (2012)
	*B. adolescentis* ATCC 15703 inhibits fracture-induced systemic inflammation	Roberts et al. (2020)
	*B. lactis* HN019 inhibits the pathogen growth	Oliveira et al. (2017)
Regulating host immune system	*B. animalis* subsp. lactis Bb-12 increased the levels of total IgA and anti-β-lactoglobulin IgA	Fukushima et al. (1999)
	*B. breve* ATCC 15700 promotes the development of regulatory T cells	Zhang et al. (2010)
	*B. animalis* subsp. lactis HN019 promoted the phagocytic activity of peripheral blood leucocytes and peritoneal macrophages	Gill et al. (2000)
	*B. longum* subsp. infantis CCUG 52486 may promote NK cell activity and cytokine production	You and Yaqoob, (2012)
Other benefits	A mixture of *B. longum* BB536 and *B. pseudocatenulatum* G4 can ameliorate cardiovascular symptoms by regulating cholesterol levels	Al-Sheraji et al. (2012)
	A mixture of *B. longum* BB536, *B. infantis* M-63, and *B. breve* M-16 V ameliorates the allergen pollen-induced rhinitis symptoms probably by modulating the host innate immunity	Miraglia Del Giudice et al. (2017)
	*B. pseudocatenulatum* CECT 7765 restores vascular dysfunction by downregulating NO release	Mauricio et al. (2017)
	*B. breve* A1 prevents cognitive impairment in Alzheimer’s disease model mice by suppressing the expressions of some specific genes	Kobayashi et al. (2017)

Additionally, multi-strain or multi-species probiotic formulations have greater efficacy in fighting infections compared to single strain administration due to the complementary or even synergistic effects of the multi-strain/species formulation (Timmerman et al., 2004; Collado et al., 2007; Chapman et al., 2011). A combination of *B. longum* subsp. infantis CECT 7210 and *B. animalis* subsp. lactis BPL6 enhances gut health and ameliorates *S. typhimurium*-infection in the porcine model (Barba-Vidal et al., 2017). A mixture of *B. longum* BB536 and *L. rhamnosus* HN001 significantly reduced potentially harmful bacteria and enriched beneficial ones (Toscano et al., 2017) in the gut microbiota. A cocktail of probiotic *Lactobacilli* and *Bifidobacteria* showed antimicrobial and anti-biofilm activities against multidrug-resistant *E. coli* (Abdelhamid et al., 2018), and pretreatment with yogurt containing *Lactobacillus acidophilus* La5 or *B. lactis* BB-12 suppresses *H. pylori* infections effectively in humans (Wang et al., 2004; Sheu et al., 2006). A fermented formula containing *B. breve* c50 and *Streptococcus thermophilus* 065 reduces the severity of acute diarrhea among healthy young infants (Thibault et al., 2004).

Aside from the common protection against pathogenic bacteria, *Bifidobacteria* also exhibit antiviral activities. For example, *B. adolescentis* SPM1605 inhibits human enterovirus Coxsackievirus B3, thus preventing the virus infection-related acute heart failure and aseptic meningitis (Kim et al., 2014); *B. longum* IBG inhibits infection by rotavirus *in vitro* and decreases the duration of diarrhoea in pediatric patients (Lee et al., 2015).

### Anticancer Activity

Probiotics have been employed to prevent and treat cancers for decades (Rowland et al., 1998; Lee et al., 2004; Paolillo et al., 2009; Ohara et al., 2010), where *Bifidobacteria* can effectively inhibit cancers in animal models. As an example, dietary supplementation of *B. longum* BB-536 significantly inhibits the 2-amino-3-methylimidazo [4,5-f]quinoline (IQ)-induced incidence of the colon (100% inhibition) and liver (80% inhibition) tumours in male rats and suppresses the IQ-induced mammary carcinogenesis (50% inhibition) and liver carcinogenesis (27% inhibition) of female rats (Reddy and Rivenson, 1993). *B. longum* BCRC 910051 prevents the development of 1,2-dimethylhydrazine-induced colonic tumorigenesis (Foo et al., 2011). An *in vitro* study shows that *B. bifidum* BGN4 inhibits the growth of several human colon cancer cell lines such as HT-29 and HCT-116 (Ku et al., 2009).

### Anti-inflammation

Inflammation is a physiological response generally triggered by damage to the living tissues. The inflammatory response is a defense mechanism that protects the host from infection and injury. *Bifidobacteria* colonize primarily in the oral cavity and intestinal tracts. They thus have been applied to suppress and prevent some oral and enteric inflammations including irritable bowel syndrome (Guglielmetti et al., 2011; Ringel-Kulka et al., 2011; O'Mahony et al., 2005), intestinal barrier functions (Krumbeck et al., 2018), and infant colic impairment (Kobayashi et al., 2019; Xiao et al., 2020). However, some strains also inhibit cutaneous inflammations, e.g., a mixture of *B. breve* M-16V and *B. longum* BB536 reduces the development of eczema and atopic dermatitis in infants (Enomoto et al., 2014).

### Enteritis

An *in vitro* study shows that *B. animalis* MB5 protects intestinal Caco-2 cells from the inflammation-associated response by counteracting neutrophil migration and partly decreasing pathogen adhesion (Roselli et al., 2006). Many animal studies also validate the anti-inflammatory potentials. For example, a combination of *B. bifidum* and *B. longum* was found to effectively prevent devastating necrotizing enterocolitis (NEC) in an animal model (Wu et al., 2013); *B. adolescentis* IM38 ameliorates high fat diet-induced colitis by inhibiting NF-κB activation and lipopolysaccharide production by gut microbiota (Lim and Kim, 2017); *B. bifidum* ATCC 29521 restores the colon mucus layer of mice with ulcerative colitis by modulating NF-κB signalling pathway and rebuilding the gut intestinal microbiome equilibrium (Din et al., 2020). *B. breve* CECT7263 attenuates endothelial dysfunction by regulating the levels of acetate and trimethylamine produced by gut microbiota (Robles-Vera et al., 2020).

Human studies show that *B. breve* M-16V potentially protects infants from developing NEC (Wong et al., 2019). Mechanistic studies indicated that M-16V can promote early gut microbial colonization, thus regulating the host immunity and preventing the inflammatory response. The combination of 2 *B. breve* strains (BR03 and B632) coupled to a gluten-free diet has shown a positive effect on decreasing the production of pro-inflammatory cytokine TNF-α in children with celiac disease (Klemenak et al., 2015). A clinical study shows that the probiotics mixture containing *B. bifidum* BGN4, *B. lactis* AD011, *L. acidophilus* AD031 and *L. casei* IBS041 can effectively relieve irritable bowel syndrome (Hong et al., 2009). A synbiotic formula composing probiotics (*B. breve* and *L. casei*) and the prebiotics galactooligosaccharides improved the intestinal absorptive function and motility of patients with short bowel syndrome (Kanamori et al., 2004; Bongers et al., 2010). The combination of *B. breve* Yakult and *L. casei* Shirota can prevent infant enterocolitis (Kanamori et al., 2010), reduce NEC incidence and improve intestinal motility in infants (Braga et al., 2011). An open-label pilot study revealed that the administration of *B. longum* BB536 effectively induced remission of patients with ulcerative colitis (Takeda et al., 2009). Three *B. breve* strains and a *B. longum* strain show potential in treating enteric disorders in newborns such as infantile colics (Aloisio et al., 2012).

### Lactose Intolerance

Lactose intolerance occurs in patients that produce insufficient lactase in the small intestine to digest dietary lactose, usually derived from dairy food. Undigested lactose flows into the colon, where the lactose is catabolized by the gut microbes, triggering the lactose intolerance symptoms, including diarrhoea, flatulence, nausea, stomach cramps, and vomiting. *B. animalis* subsp. animalis IM386 ameliorates diarrhoea and flatulence in lactose-intolerant individuals (Roškar et al., 2017) because it facilitates lactose degradation in the small intestines. Mixed probiotics containing *B. animalis* subsp. animalis IM386 and *L. plantarum* MP2026 also alleviated some gastrointestinal symptoms in lactose-intolerant subjects (Roškar et al., 2017).

### Constipation

Constipation usually results from changes in diet or inadequate intake of fibre, where treatment using a multi-component probiotics formula consisting of *B. bifidum*, *B. infantis*, *B. longum*, *L. casei*, *L. plantarum,* and *L. rhamnosus* has positive effects on alleviating symptoms of constipation (Bekkali et al., 2007). *B. longum* W11 was also found to relieve the constipation symptoms of patients with irritable bowel syndrome, synergize with rifaximin as an adjuvant antibiotic treatment, and treat minimal hepatic encephalopathy (Di Pierro and Pane, 2021).

### Oral Inflammation

Aside from preventing enteric inflammations, *Bifidobacteria* show some inhibitory effects on oral inflammations. *B. lactis* DN-173010-fermented yogurt fed to patients elicit a positive effect on gingival inflammatory parameters (Kuru et al., 2017) because it can decrease both concentration and the total amount of IL-1β in gingival crevicular fluid. Oral administration of *B. lactis* HN019 as an adjunct potentiates the effects of scaling and root planing (SRP) in treating experimental periodontitis in rats (Ricoldi et al., 2017) and patients (Invernici et al., 2018).

### Promoting Psychological Health

Psychobiotics are a category of probiotics that confers mental health benefits, and many *Bifidobacteria* are functionally considered psychobiotics. Studies show that the gut microbiota of stress-resilient mice has lower *Bifidobacteria* than control and susceptible mice, and supplementation of *Bifidobacteria* to the susceptible mice significantly increased the resilience compared with vehicle-treated mice (Yang et al., 2017a). These findings suggest that *Bifidobacteria* may confer resistance to stress. *B. breve* CCFM1025 showed antidepressant-like effect in chronically stressed mice due probably to the capacity of utilizing various carbohydrates and producing neuroactive metabolites, such as tryptophan, hypoxanthine, and nicotinate (Tian et al., 2021). *B. breve* CCFM1025 can also reverse chronic stress-induced depressive symptoms (Tian et al., 2020). *B. adolescentis* 150 shows anti-depression properties when fed to mice (Yunes et al., 2020) due to the ability to produce gamma-aminobutyric acid, a neurotransmitter inhibitor of the central nervous system (Dinan et al., 2013). *B. adolescentis* NK98 alleviates anxiety/depression symptoms through regulating gut immune responses and microbiota composition (Jang et al., 2019). *B. adolescentis* IM38 can attenuate anxiety by regulating the benzodiazepine site of the GABA_A_ receptor and modulating stress-related cytokine expression (Jang et al., 2018). *B. longum* 1714 reduces stress, anxiety and depression-related behaviours in anxious mice (Allen et al., 2016). The potential probiotic *B. pseudocatenulatum* CECT 7765 ameliorates depression comorbid with obesity via regulating endocrine and immune mediators of the gut-brain axis (Agusti et al., 2018). *B. breve* 1,205 reduces general anxiety behaviours in mice (Savignac et al., 2014). The combinatorial use of *Lactobacillus helveticus* R0052 and *B. longum* R0175 has anxiolytic-like activity (Messaoudi et al., 2011a) and reduces post-myocardial infarction depression symptoms (Arseneault-Bréard et al., 2012) in rats.

Various clinical trials have further supported these claims on the psychological health promotion effects. For instance, the oral administration of *B. longum* NCC3001 to depression patients reduces depression scores and alters patients’ brain activity (Pinto-Sanchez et al., 2017). *B. longum* 1714™ was found to modulate brain activity by regulating resting neural activity and neural responses (Wang et al., 2019a). *B. longum* 1714 modulates electrophysiology and neurocognition in healthy humans (Allen et al., 2016). The mixed probiotics of *B. longum* and *L. helveticus* significantly reduced the depressive symptoms of patients with Major Depression Disorder (Kazemi et al., 2019), presented beneficial psychological effects in healthy human volunteers (Messaoudi et al., 2011a), and decreased stress-induced gastrointestinal discomfort (Messaoudi et al., 2011b).

### Decreasing Fat Accumulation

Obesity is a complex disease and increases the risk of other diseases and health problems, such as heart disease, diabetes, high blood pressure and certain cancers. Some *Bifidobacteria* probiotics can reduce the host fat accumulation. *B. lactis* CECT 8145 reduces fat content and modulates lipid metabolism and antioxidant response in *Caenorhabditis elegans* (Martorell et al., 2016). Another study using heat-treated *B.* lactis CECT8145 found an increased lean mass and ameliorated metabolic syndrome in cafeteria-fed obese rats (Caimari et al., 2017). Similar studies using the same probiotic also show that administering either living or heat-treated *B. lactis* CECT8145 can reduce anthropometric adiposity biomarkers linked to changes in host immune system regulation and enrichment of *Akkermansia* genus in the gut of abdominally obese individuals (Pedret et al., 2019). *B. lactis* 420 is another strain found to reduce fat mass and glucose intolerance in both obese and diabetic mice (Stenman et al., 2014) by reducing the translocation of gut microbes.

### Facilitating the Host Nutrition Absorption

Consumption of probiotics has been reported to facilitate the absorption of nutrients, e.g., vitamins and calcium ions (Ballini et al., 2019). *B. longum* BB536 can increase the abundance of nutrients including pimelate, biotin and butyrate, by facilitating the fermentation processes resulting from the microbial crosstalk between *B. longum* BB536 and human gut-derived microbiota (Sugahara et al., 2015). Additionally, *B. pseudocatenulatum* is commonly found in human faecal samples throughout their lifetime, where some of these strains have shown beneficial properties, such as the production of enterolignan, urolithin, and conjugated linoleic acid (Yang et al., 2017b; Vickers, 2017; Gaya et al., 2018).

### Promoting Bone Health

Osteoporosis, a common bone metabolic disorder caused by low bone mass and deterioration of the bone tissue, results in the individual being prone to fractures. Using probiotic *B. longum* ATCC 15707 (Parvaneh et al., 2015) or in combination with yacon flour (Rodrigues et al., 2012) can increase bone mass density by elevating the expression of *Sparc* and *Bmp-2* genes. Additionally, *B. adolescentis* ATCC 15703 can modulate bone repair by dampening fracture-induced systemic inflammation (Roberts et al., 2020), and the topical use of *B. lactis* HN019 promotes a protective effect against alveolar bone (Oliveira et al., 2017).

### Regulating the Host Immune System

These probiotics can regulate the host immune system for the amelioration or prevention of diseases. *B. animalis* subsp. lactis BB-12 was found to protect murine pups and dams from exposure to food antigens by increasing total IgA and anti-β-lactoglobulin IgA levels in fecal extracts (Fukushima et al., 1999). *B. breve* ATCC 15700 suppresses the skewed T helper 2 pattern responses by promoting Treg development (Zhang et al., 2010). *B. animalis* subsp. lactis HN019 can enhance natural immunity in healthy elderly subjects by increasing the anti-inflammatory cytokine IFN-α and phagocytic activity (Arunachalam et al., 2000). HN019 also enhances several natural and acquired immunity indices in healthy mice, including NK-cell activity, IFN-γ production, antibody responses to antigens, and the phagocytic activity of peripheral blood leukocytes and macrophages (Gill et al., 2000). *L. helveticus* Bar13 and *B. longum* Bar33 can synergistically improve the physiologic status and immunity of older adults by increasing regulatory T (Treg and Tr1) cells and decreasing γδ T cells (Finamore et al., 2019). *In vitro* studies show that *B. bifidum* BGN4 can activate differentiation of host macrophages and stimulate the production of IL-10 and IL-6 (Lee et al., 2002; Kim and Ji, 2006). *B. longum* subsp. infantis CCUG 52486 shows strong immunomodulatory potential comparable with well-known commercial strains (e.g., *B. longum* SP 07/3, *L. rhamnosus* GG and *L. casei* Shirota) based on the IL-10/IL-12 ratios (You and Yaqoob, 2012).

### Other Beneficial Effects

A combination of *B. longum* BB536 and *B. pseudocatenulatum* G4 ameliorates cardiovascular symptoms by decreasing total cholesterol, LDL-cholesterol, triglyceride levels, malondialdehyde, and increasing HDL-cholesterol levels concentrations (Al-Sheraji et al., 2012). A *Bifidobacteria* mixture of *B. longum* BB536, *B. infantis* M-63, and *B. breve* M-16 V prevents allergen pollen-induced rhinitis symptoms (Miraglia Del Giudice et al., 2017). *B. lactis* BB-12 alters the colonization of cariogenic bacteria, prevents dental caries (Çaglar et al., 2008) and decreases plaque and gingival indexes (Toiviainen et al., 2015). *B. pseudocatenulatum* CECT 7765 can restore the obesity-induced vascular dysfunction by reducing nitric oxide release (Mauricio et al., 2017) and prevent gut-derived complications in experimental chronic liver disease via maintaining gut homeostasis (Moratalla et al., 2016). *B. breve* A1 exhibits therapeutic potential for preventing cognitive impairment in Alzheimer’s disease model mice by suppressing the hippocampal expressions of amyloid-β-induced specific genes (Kobayashi et al., 2017). While the studies on *Bifidobacterium* as probiotics may be considered extensive, it is inevitable that with the latest multi-omics approaches, more strains of *Bifidobacteria* and its numerous therapeutic activities are soon to be discovered in future with the advent of highly efficient isolation and determination methods.

## Current Development of Engineering of Bifidobacterium

### Bifidobacterium as a Chassis

#### *Bifidobacterium* Biochemical Properties and Currently Used Chassis for Genetic Engineering

The Gram-positive *Bifidobacterium* is an anaerobic branched rod-shaped actinobacterium often associated with symbiotic bacterial-host relationships with mammals, particularly humans. Given the anaerobic nature of the microbe, the natural microbial growth rate is relatively slower than their other counterparts in the microbiota. Thus, *Bifidobacterium* is usually administered at a high cell count to overwhelm the microbiota and displace the pathogens in the host to elicit its therapeutic properties.

Microbes from the Actinobacteria phyla, including *Bifidobacterium*, are generally recognized as natural product producers with the basic biochemical makeup for producing valuable metabolites used in pharmaceuticals, agricultural, environmental, and industrial applications (Hazarika et al., 2020). The use of *Bifidobacterium* as a chassis for genetic manipulation requires a better understanding of microbial biochemistry. Understanding the pathways regulating microbial behaviour in synthetic biology and metabolic engineering allows streamlining cellular processes to elicit the appropriate responses. These biochemical attributes include producing polyketides, short-chain fatty acids, conjugated linoleic acid, and metabolizing fructose, lactose, and cholesterol.

##### CoA Derivative Production

**FIGURE 2:**
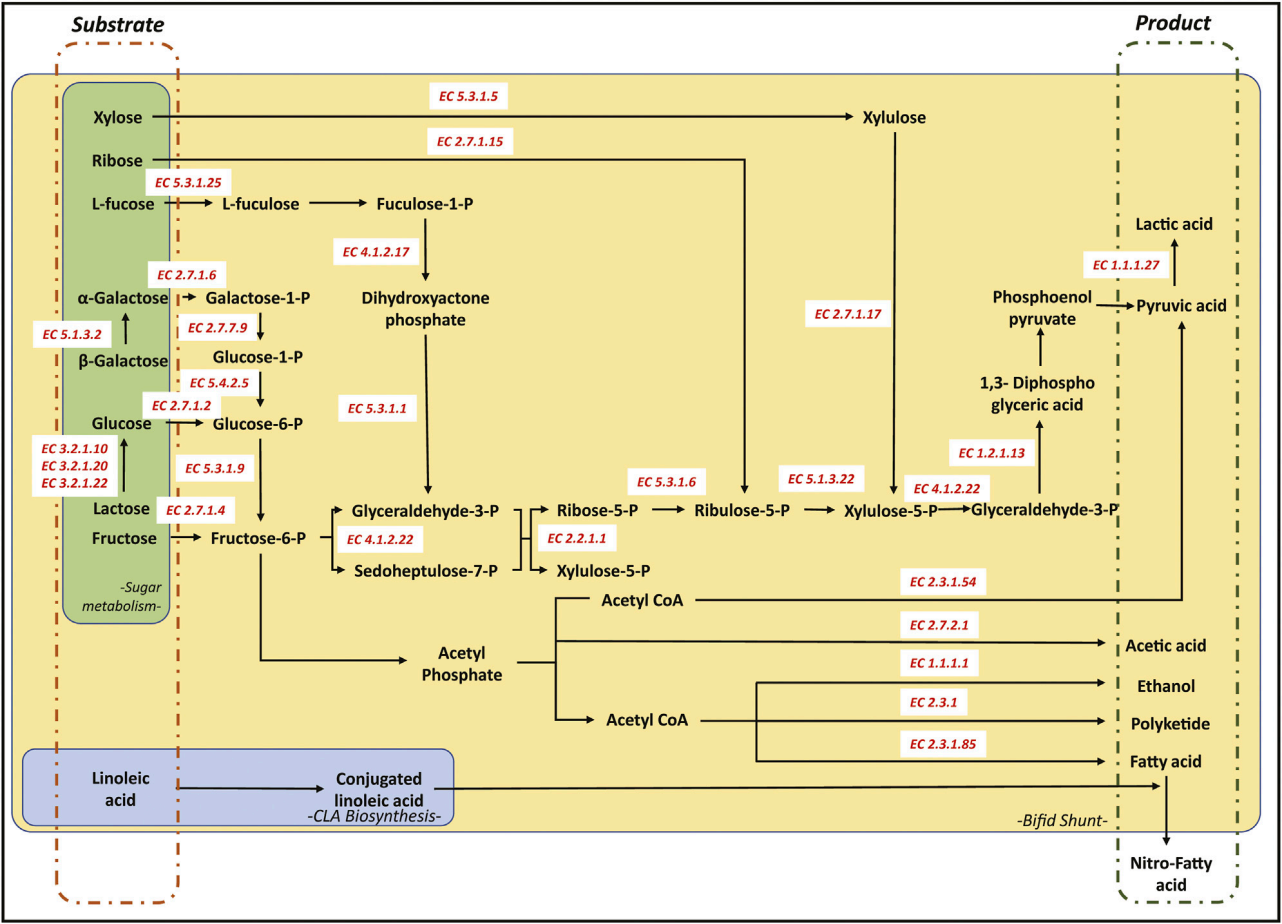
Schematic representation of the Bifid shunt pathway (yellow), CLA pathway (blue) and other carbohydrate degradation pathways (green). *All corresponding enzymes in the pathway were annotated using the Enzyme Commission numbers as recommended by the Nomenclature Committee of the International Union of Biochemistry and Molecular Biology (IUBMB).*

*Bifidobacteria* can be considered a powerful workhorse for producing polyketide products. This ability can be attributed to the Bifid shunt that can effectively produce more CoA derivatives than other conventional production methods of CoA derivatives (Wang et al., 2019b). The acetyl-CoA, malonyl-CoA, and other CoA derivatives are the building blocks involved in the biosynthesis of polyketide, fatty acids, butanol, isoprenoids, and amino acids (Figure 2) (O’Callaghan and Van Sinderen, 2016). Additionally, some *Bifidobacteria* can metabolize the byproducts and waste of the host body to produce a higher pool of CoA derivatives, providing these microbes with an additional edge in generating a larger pool of CoA derivatives for the biosynthesis of value-added chemicals. For instance, *B. animalis* subspecies lactis was found to degrade oxalate (a cellular byproduct secreted by the host cells in the amino acid metabolism), providing the microbe with a higher pool of oxalyl CoA while reducing the risk of oxalate toxicity to the host (Turroni et al., 2010). There have been efforts to use these probiotic strains to treat patients with weak kidney functions and at high risk of developing kidney stones. Another study investigating the role of *Bifidobacteria* in producing short-chain fatty acid (SCFA) further revealed that these microbes could metabolize complex sugar such as glucomannan to produce higher pools of SCFA, including lactic, acetic, propionic, and butyric acid (Usta-Gorgun and Yilmaz-Ersan, 2020).

The produced CoA derivatives are used to produce secondary metabolites by modular enzymes encoded in the *Bifidobacterium* gene (Figure 2). The gene sequences of various *Bifidobacterium* revealed several polyketide synthase (PKS) gene clusters, including those from the Type 1 and Type 3 PKSs. A study comparing the *Bifidobacteria* isolated from humans and marmosets showed that Type 1 PKS was conserved across *Bifidobacteria*, including *B. myosotis, B. reuteri, B. breve, B. longum, B. tissieri, B. bifidum,* and *B. callitrichos* (Brown et al., 2019). Closer investigation on the metabolomics of *B. breve* UCC2003 showed that under the presence of high bile salt concentrations, the *Bifidobacteria* reverts to the sessile biofilm state through the expression of polyketide synthase Bbr_0,204/0,205. The study hypothesized the role of PKS in the microbe’s adaptive behaviour in the presence of bile salts (Kelly et al., 2020).

Like polyketide production, the SCFAs, medium-chain fatty acid (MCFA), and fatty acid ethyl esters are produced through the fermentation of complex sugars in *Bifidobacteria* to generate the various CoA-derivatives (Figure 2). These SCFAs produced include butyrate, propionate, and so forth that play an essential role in host health, including boosting the metabolism and reducing the risk of developing diabetes (Usta-Gorgun and Yilmaz-Ersan, 2020). These SCFAs are generated as a byproduct of the Bifid shunt and can be used for the biosynthesis of MCFA using a homologous protein to polyketide synthases, known as fatty acid synthase (FAS) (Gu et al., 2016). FASs are modular enzymatic proteins that catalyze the CoA derivatives to produce the various fatty acids.

Thus, it is evident that the presence of the Bifid shunt and the various metabolic processes within *Bifidobacterium* strains provides the necessary metabolic precursors needed for engineering PKS, FAS and other CoA related pathways. This unique characteristic facilitates the biosynthesis of value-added compounds through the use of multi-domain modular enzymes. So far, there has been no reported use of *Bifidobacterium* in the bioproduction of polyketides or fatty acids due to the limited gene engineering tools. However, leveraging this attribute would help improve the productivity of the bioconversion (the substrate/product ratio).

##### Conjugated Linoleic Acid

Many *Bifidobacteria* are natural producers of conjugated linoleic acids (CLA), resulting from the biohydrogenation of linoleic acid (LA) and other unsaturated fatty acids (Figure 2). The first reported strain that produces CLA is *B. breve* with a 66% endpoint conversion, where the converted LA to CLA was secreted into the surrounding medium (Park et al., 2011). Other strains showing a similar ability to convert LA to CLA includes *B. infantis, B. dentium, B. catenulatum and B. pseudocatenatalum* (Raimondi et al., 2016)*.* The biosynthesis of CLA from LA involves a three-step process, relying on LA isomerase enzymes, direct reduction of the unstable variant, and delta-9 desaturase enzyme (Coakley et al., 2003). While CLA is directly linked to many health benefits, the CLA is a preferred substrate for producing nitrated fatty acids (Bonacci et al., 2012). The various CLA health benefits include anti-inflammation, antiinfection, anticancer, and increasing host metabolism. On the other hand, nitro-fatty acids are often used as an agent to reduce blood pressure and prevent inflammation and other fibrotic diseases (Schopfer et al., 2018). The natural ability of *Bifidobacterium* to convert and produce CLA generates a perpetual pool of CLA for the biosynthesis of nitro-fatty acids and their *B. animalis* subsp. lactis various derivatives.

##### Fructose and Lactose Metabolism

Fructose is often linked to various metabolic and heart diseases, where increased dietary levels of fructose are linked to an increased risk of intestinal inflammation (Tan et al., 2021). Thus, probiotic strains that can absorb and utilize fructose as a carbon source is considerably preferred. Conversely, various *Bifidobacterium* strains have been shown to possess fructokinase (fructose phosphotransferase) activity, crucial for fructose metabolism (Figure 2) (Mazé et al., 2007). The phosphorylated fructose (fructose-1-phosphate) is then assimilated into the Bifid shunt to increase CoA derivatives production. This fructokinase activity is encoded by the *fruA* gene in *B. breve* UCC2003 that is homologous to the *EIIBCA* gene of the phosphoenolpyruvate: sugar phosphotransferase system of *B. longum* NCC2705. Both the encoded FruA and EIIBCA are known to play an integral part in the breakdown and assimilation of fructose (Mazé et al., 2007).

Similarly, many people suffer from lactose intolerance, where symptoms can range from mild indigestion to severe water loss and malnutrition. To this extent, the ability of *Bifidobacterium* to metabolize lactose can be considered as a potential health benefiting property. Additionally, lactose utilization should not produce biochemicals that are toxic to the human host. Interestingly, studies have shown that *Bifidobacterium* has a higher preference for lactose than other simple sugars like glucose. For instance, in *B. longum* NCC2705, the gene *glcP,* a putative glucose transporter, showed that the presence of lactose reduces the influx of glucose into the *Bifidobacterial* cytosol conferring a preference to lactose instead (Parche et al., 2006). Additionally, studies using *B. bifidum* indicate that the microbe presents some lactase activity in a slightly acidic environment within the gastrointestinal tract, resulting in the breakdown of lactose to glucose and galactose that are then assimilated into the Bifid shunt (Passerat et al., 1995).

Similar to the observations discussed earlier, the intake of the various sugars fructose and lactose is highly dependent on the concentrations of other sugars in the surrounding environment. The synergistic interactions of the various sugars result in a change in the preference of certain sugar types that enters the Bifid shunt. In some instances, such as in *B. adolescentis* MB239, the *Bifidobacterium* prefers the uptake of fructose and lactose depending on other types of sugars where the studies suggest some form of synergistic interactions between the sugars (Amaretti et al., 2006; Tan et al., 2021).

#### Strain Optimization of *Bifidobacterium*

However, not all strains of *Bifidobacterium* have the desired properties of being a probiotic strain. A probiotic strain needs several attributes, including the ability to survive the human host environment and provide host-benefiting properties while showing no pathogenicity to the host. The following subsection discusses the limitations of using *Bifidobacterium* as an engineering chassis and methods of circumventing these limitations.

##### Bile Salt Intolerance

Some studies found that certain *Bifidobacterium* could not survive in the presence of bile salt due to the lack of conjugated bile salt hydrolase activity. In particular, the presence of glycoconjugate bile salt such as glycodeoxycholic acid presents higher toxicity to *Bifidobacterium* under an acidic environment. A study concluded that the susceptibility of *Bifidobacteria* to the glycoconjugated bile salt is dependent on the conjugated bile salt hydrolase activity (CSBH) (Grill et al., 2000). The presence of glycodeoxycholic and taurodeoxycholic acids was found to interfere with the survivability of the *B. animalis* ATCC25527*, B. breve* ATCC15700*, B. longum* ATCC15707 *and B. coryneforme* under lower pH conditions. These bile salt-sensitive *Bifidobacteria* are either CBSH or lacking the deconjugating properties, where either one of the two properties allows the *Bifidobacteria* to protonate and deconjugate the bile salt prior to export to the surrounding (Grill et al., 2000). Currently, many variants of *Bifidobacteria* naturally resistant to bile salts are considered good engineering chassis. However, should there be a need to develop a novel strain of *Bifidobacterium* that does not have the bile salt tolerance, engineering these microbes to have CSBH activities can be considered an excellent strategy to engineer the microbial cell.

##### Mucin Degrading Bifidobacterium

The mucin layer forms a protective barrier system crucial for preventing the adhesion and penetration of pathogens, toxins, and other damaging agents in the gut. The mucin layer comprises highly glycosylated *O*-linked glycoproteins secreted primarily by the exocrine glands and mucosa (Karav et al., 2018). The protection conferred by the mucin layer is attributed to the glycoprotein structure within the mucus layer that retains a large body of water (>95%), where the retain water forms a reservoir of electrolytes, antibodies, and nucleic acids. Mucin degradation is commonly facilitated by mucolytic taxa such as those from the *Bacteroides* that carries a large variety of enzymes, including proteases, sulfatases, fucosidases, neuramidases, β-galactosidases, α-*N*-acetylgalactosaminidases, α-*N*-acetylglucosaminidases, and exo/endo-β-*N*-acetyl-glucosamindases (Karav et al., 2018). Moreover, the depleted mucin layers might result in the translocation of the gut microflora and other toxins across the exposed gut lining tissues (Abe et al., 2010). Several intestinal *Bifidobacterium* isolates can degrade the mucin as a form of sustenance through *endo*-α-*N*-acetylgalatosaminidase and 1,2-α-fucosidase (Ruas-Madiedo et al., 2008). A study conducted on 22 different *Bifidobacterium* strains isolated from the human host found that most *B. longum* and *B. bifidum* isolates can break down the mucin layers in the host gut (Ruas-Madiedo et al., 2008). Conversely, the use of non-mucin degrading variants of *Bifidobacterium* such as *B. longum* subsp. *infantis* EVC001 showed that the probiotic strain prevents colonic mucin degradation in breastfed infants. This characteristic of the probiotic strain is due to the displacement of other mucin-degrading *Bacteroides* population (Karav et al., 2018). While there is limited proof to support the adverse effects of mucin-degrading *Bifidobacterium,* it is preferable that the engineered *Bifidobacterium* does not have the mucin-degrading capabilities as it would be deemed safer for consumption. The selection of *Bifidobacterium* as a suitable engineering host could leverage on naturally occurring *Bifidobacteria* or through knockout of the corresponding mucin-degrading genes.

#### Current Bifidobacterium Used as Engineering Chassis

Numerous studies have used *Bifidobacterium* as an engineering chassis, mainly to respond to various environmental triggers. These engineered *Bifidobacterium* have been designed to facilitate various roles, such as biosensing (Cronin et al., 2012), treatment of diseases (Cronin et al., 2012) or improved bioprocessing. Other approaches have been used mainly to elucidate the *Bifidobacterium* cellular function and improve microbial tolerance and survivability in the environment (Watson et al., 2008; He et al., 2012a). An example of conventional *Bifidobacterium* engineering chassis is *B. breve* UCC 2003, which was used for improving tolerance to bile salt and survival under the gastrointestinal tract physiological environment. This improved tolerance was achieved by expressing the *Listeria monocytogenes* bile resistance gene, *BilE* (Watson et al., 2008)*.* A similar *Bifidobacterium* was engineered to sense tumours and set the stage for later anticancer treatment using the microbial cell line (Cronin et al., 2012). Another commonly used strain of *Bifidobacterium* is the *B. longum* 105-A that was previously used in investigating the inducible and constitutive promoters. The isolated promoter sequences were reintroduced into *B. longum* 105-A with the α-galatosidase reporter gene (Sakanaka et al., 2014). This particular strain is easier to manipulate compared to other variants of *Bifidobacterium* due to the higher transformation efficiency used primarily in knocking out/down genes for closer study of the biochemical pathway (Kanesaki et al., 2014). Similar to *B. breve* UCC 2003, *B. longum* 105-A was also used in improving its tolerance to oxidative stress through the expression of the *katE* catalase gene isolated from *Bacillus subtilis* (He et al., 2012a)*.* Another study used a similar approach to confer oxidative stress tolerance in *B. thermophilum* RBL67 through transferring the *B. longum* gene *bl_1,404* (Stevens et al., 2017). The various *Bifidobacterium* used as an engineering chassis, their culture collection bank, and their source are described in Table 2.

**TABLE 2:** The various *Bifidobacterium* used as an engineering chassis.

Species	Microbial bank	Source	Ref
*Bifidobacterium breve* UCC2003	UCC culture collection		Watson et al. (2008), Cronin et al. (2012)
*Bifidobacterium thermophilum* RBL67	ECACC General Collection	Baby faeces isolate	Stevens et al. (2017)
*Bifidobacterium longum* subspecies *longum* DSM20219	DSMZ	Adult intestinal isolates	
*Bifidobacterium longum* NCC2705 HPR2	Nestle Research Center	Peroxide resistant mutant derivative of NCC2705	Oberg et al. (2015)
*Bifidobacterium* subspecies *infantis* DSM20088	DSMZ	Adult intestinal isolates	Landete et al. (2014)
*Bifidobacterium longum* Reuter 1963 CECT 4551	CECT	Infant intestine	
*Bifidobacterium breve* INIA P734	INIA	Adult intestinal isolates	
*Bifidobacterium animalis* subsp. *animalis* JCM 1190/ATCC 25527	Riken JCM Catalogue/ATCC	Rat feces	Sakanaka et al. (2014)
*Bifidobacterium breve* 203	In-house	Human feces	Nunoura et al. (1997), Landete et al. (2014)
*Bifidobacterium longum* 105-A	In-house	Human feces	Ruas-Madiedo et al. (2008); He et al. (2012a); Kanesaki et al. (2014)

### Types of Genetic Regulatory Tools

Genetic tools used to regulate the Bifidobacterial gene expression could be generally divided into the promoters, ribosomal binding sites (RBS), and terminator sequence. The various elements regulating gene expression will be discussed in the following sections. These various elements can be used individually or concertedly to optimize the regulated microbial function.

#### Promoter Sequences for Heterologous Gene Expression

Promoters are gene sequences that recruit the RNA polymerase to trigger mRNA transcription for downstream cellular processes. These sequences are located upstream of the coding region, including the ribosomal binding site and the gene sequences. The naturally occurring promoter sequences contains promoter core motifs located at the -35 to -10 region from the start of the coding sequence. These sequences conventionally contain the TTGNNN and the TANNNT conserved sequences respectively (Kozakai et al., 2021).

Promoter sequences are generally divided into two categories, namely the constitutive promoters and the inducible promoters. Constitutive promoters piggyback on the cellular metabolic function of the *Bifidobacterium*, facilitating a gene expression depending on the microbial metabolism. On the other hand, inducible promoters often carry a repressor/activator protein that binds on the operator site at either side of the -35 sequence. The repressor protein will bind on the operator site, inhibiting the recruitment of the RNA polymerase complex. In the presence of the corresponding ligand, binding of the ligand to the repressor causes conformational changes triggering the release from the operator site. On the other hand, activators will bind on the operator site after binding to the target ligand, facilitating the recruitment of the RNA polymerase complex.

Various constitutive promoters for *Bifidobacterium* expression were developed by sifting through the gene sequences within the *Bifidobacterium* genome (Sakanaka et al., 2014; Kozakai et al., 2021). The promoter core motif sequences were identified *in silico* using the hidden Markov model on the upstream sequences of the transcriptional start site of various coding genes in *Bifidobacterium.* This approach was used to identify the various putative constitutive promoter sequences in *B. longum* NCC2705 (Kozakai et al., 2020; Kozakai et al., 2021) and *B. longum* 105-A (Sakanaka et al., 2014). Additionally, the space between the -35 and -10 sequences can range from 11 to 18 nucleotides in length, where a shorter space length was found to improve the transcription levels (Kozakai et al., 2020).

Inducible expression within *Bifidobacterium* is mainly linked to the sugar-related inducers that are linked to the Bifid shunt. Some of these promoters were found to function in other microbes, whereas others were specific to *Bifidobacterium.* Methods of identifying these inducible promoters are through transcriptomic analysis of genes that are upregulated in the presence of the inducer molecules, where through annotating the upregulated genes, the corresponding promoter region is investigated. This approach identified sucrose and raffinose inducible promoters from *B. lactis* (Trindade et al., 2003) and *B. longum* NCC2705 (Kullin et al., 2006). Another example would be using the pNZ8048 nisin inducible promoter initially used for expression within *Lactobacillus* (Landete et al., 2014)*.* In a similar study, the elongation factor Tu from *B. longum* subsp. *infantis* ATCC15697 was used for stable anaerobic expression of green fluorescent protein in both *B. longum* Reuter 1963 CECT4551 and *B. breve* INIA P734 (Landete et al., 2014).

In order to facilitate a better understanding of the various working promoters and the −35 and −10 sequences, Table 3 provides the information of the various types of promoters, the source plasmids, sequence origin and the promoter motifs.

**TABLE 3:** The list of promoter sequences used in *Bifidobacterial* engineering.

		Promoter motif					
Plasmid	Promoter	-35 sequence	Space (nt)	-10 sequence	Inducible/constitutive	Notes	Ref
pBFS46	P_*gap_Blo*_	TTGCCA	18	TACAGT	Constitutive	Isolated from *B.longum* 105-A genome	Sakanaka et al. (2014)
pBFS48	P_*xfp_Blo*_	AAGTCG	14	CATGAC	Constitutive		
pBFS52	P_*xfp_Bbr*_	AAGTCA	14	CATGAT	Constitutive	Isolated from *B.breve* 203 genome	
pBLHU15	P_*hup*_	TTCGCA	15	TATCAT	Constitutive	Isolated from *B. longum* ATCC15707	Takeuchi et al. (2002)
pLFB1012/pBCMAT	P_*gap*_	TTGCCA	18	TACAGT	Constitutive	Isolated from *B.longum* DSM20088 genome	Stevens et al. (2017); Kozakai et al. (2021)
pBCMAT	P_*groES*_	TTGGCA	18	TACGAT	Constitutive	Isolated from *B.longum* NCC2705 genome	Kozakai et al. (2021)
	P_*rpmB*_	TTGCGG	17	TATATT	Constitutive		
	P_*rpmH*_	TTGACT	18	TACTTT	Constitutive		
	P_*BLt43*_	TTGCGA	17	TACTAT	Constitutive		
	P_*rplU*_	TTGATT	17	TAGATT	Constitutive		
	P_*tuf*_	GTGGCA	18	TAGAAT	Constitutive		
	P_*rplM*_	TTGCCC	17	TATACT	Constitutive		
	P_*BL1230*_	TTGTGA	17	TACAAT	Constitutive		
	P_*BL1769*_	TTGACA	17	TATCAT	Constitutive		
pBFS47	P_*scrP_Blo*_	TGGACA (5/6 nt)	18	TAATAT (4/6 nt)	Carbohydrate-inducible	Isolated from *B.longum* 105-A genome	Sakanaka et al. (2014)
pBFS49	P_*fruEKFG_Blo*_	TTGAAC	17	TATAAA	Carbohydrate-inducible	Isolated from *B.longum* 105-A genome	
pBFS50	P_*cscBA_Blo*_	TTGACG	17	CATAAT	Carbohydrate-inducible	Isolated from *B.longum* 105-A genome	
pBSF51-1	P_*scrP_Ban*_	TTGCGT	17	TAAAAC	Carbohydrate-inducible	Isolated from *B.animalis* subsp. *animalis* JCM1190^T^ 105-A genome	
pBFS45-3	P_*Aga135*_	NA	NA	NA	α-Gal galactose inducible	*Promoterless inducible expression*; RBS: CCCAAGGAGTGCCT	
pNZ8048	P_*nisA*_	GGTAAT	14	ATTATA	Nisin inducible		Landete et al. (2014)
pNZ.Tu	P_*Tu*_	GCGCCA	14	GGACAA	Elongation factor Tu (anaerobic inducible expression)		

*NOTE: NA, annotates Not Available.

#### Ribosomal Binding Site Sequence Optimization Protein Translation

In the efforts to optimize the RBS for efficient protein, two primary considerations are taken into account. First, the flanking sequences of the RBS site influence the recruitment of the ribosomal subunits to the mRNA sequences (Fukiya et al., 2018). Second, the optimal distance between the RBS site and the start codon influences the rate of protein expression (Fukiya et al., 2018). Interestingly, certain studies have shown that the predominant conserved Shine-Dalgarno sequence in *Bifidobacterium* differs from conventional microbes where the most common of 6-mer consensus RBS in *B. longum* is AAGGAG as compared to the common AGGAGG (He et al., 2012b; Kozakai et al., 2020). Additionally, the space between the RBS and the start codon was found to be optimal at 5 nucleotides apart, where fewer than 5 nucleotides apart result in translation repression. While 5 nucleotides space showed the best expression outcome, changes in expression level were minimal when the RBS is 6–9 nucleotides from the start codon (He et al., 2012b).

#### Terminator Sequence Selection for Terminating Transcription Processes

In investigating the various terminators used in *Bifidobacterium,* the gene sequences were identified using WebGeSTer DB terminator database (Kozakai et al., 2021). These include the canonical (L-shaped hairpin structure) and non-canonical (I-, U-, V-, and X-shaped hairpin structures) terminators used across different phyla of microorganisms (Mitra et al., 2011). Other approaches rely on pre-existing terminator sequences found in the *Bifidobacterial* genome.

### Applications of Engineered *Bifidobacteria*

Although wild-type *Bifidobacteria* have exhibited many therapeutic applications in treating diseases (Section 1), they are endowed with more functions via synthetic biology tools such as integrating new pathways or modifying the original metabolic pathways. These engineered *Bifidobacteria* are wildly applied to treat inflammatory diseases. For example, *B. longum* HB15 expressing α-melanocyte-stimulating hormone (α-MSH) was used to combat ulcerative colitis (Wei et al., 2016a). α-MSH is a tridecapeptide that exhibits anti-inflammatory properties by regulating the production of inflammatory mediators. *B. longum* NCC2705 expressing interleukin-12 was used to treat Coxsackie virus B3-induced myocarditis in mice (Yu et al., 2012). The oral administration of *B. longum* NCC 2705 expressing oxyntomodulin can reduce food intake, body weight and plasma lipid level in overweight mice (Long et al., 2010). Oxyntomodulin is a gut hormone that reduces food intake and body weight. *B. longum* HB25 expressing the antibacterial peptide LL-37 was used for treating bacterial diarrhea (Guo et al., 2017).

Additionally, since *Bifidobacteria* can germinate and proliferate in the hypoxic regions of solid tumors (Yazawa et al., 2000; Yazawa et al., 2001; Cronin et al., 2010), *Bifidobacteria* are currently widely applied as an *in situ* delivery and production of various anticancer agents for treating tumors (Taniguchi, 2021). For example, *B. longum* 105-A was used to deliver cytosine deaminase that catalytically converts the non-toxic prodrug 5-fluorocytosine to the anticancer drug 5-fluorouracil, to the rat mammary tumors (Taniguchi et al., 2016) or mice metastatic breast tumors (Fujimori, 2006). A similar approach using *B. breve* I-53-8w was used to deliver cytosine deaminase to mice lung cancer tumors (Zu and Wang, 2014). Other approaches uses *B. longum* NCC2705 to express tumstatin protein (a powerful angiostatin that inhibits proliferation and induces apoptosis of tumorous vascular endothelial cells), used as an antitumor therapy in tumor-bearing mice (Wei et al., 2016b).

## Summary and Future Perspective

*Bifidobacterium* is considered a vital composition of the probiotic mix that essentially has various health benefiting properties when administered at the appropriate dosage. The commercial use of the various types of these probiotics have been found to exert various therapeutic properties including antiinfection, antiinflammation, anticancer, promoting host psychological and physical health, and regulating host immune system.

On the other hand, given the nature of *Bifidobacteria* and their natural biochemical properties, the microbe presents a suitable host for cellular engineering. The engineering of *Bifidobacterium* can facilitate the increased bioproduction of value-added chemicals while consuming lesser resources compared to other microbial workhorses. One of the vital biochemical processes is the Bifid shunt that produces higher CoA precursors for the bioproduction of polyketide products and fatty acid biosynthesis (Wang et al., 2019b). On top of this, the probiotic microbe readily consumes other forms of sugars that further expands the ability of the microbial cell to function as a microbial cell factory (Mazé et al., 2007; Parche et al., 2006). Additionally, with the natural ability of the microbe to produce conjugated linoleic acids (Park et al., 2011), the microbe can be used in general to produce nitro-fatty acids that have recently been gaining interest as a potential treatment for metabolic diseases (Bonacci et al., 2012). Furthermore, coupled to the natural therapeutic properties of the microbial cell, the use of *Bifidobacteria* as an engineering chassis presents an interesting alternative for metabolic engineers, synthetic biologists, and evolutionary biologists to develop *in-situ* treatment of various ailments in the human host.

However, as earlier discussed, not all *Bifidobacterium* is considered a probiotic strain due to the lack of microbial resistance to the harsh environment within the human host. Further, some of the microbes are considered unsuited for use as a probiotic owing to the microbe’s ability to break down the host mucin layers (Ruas-Madiedo et al., 2008). The mucin layer essentially functions as a protective layer within the human host, preventing the infiltration of other pathogens and the absorption of toxic compounds by the human host. Thus, to increase the list of engineerable *Bifidobacterium*, supplementing or removing certain genes would help develop better-suited probiotics for the human host.

Current known *Bifidobacterium* chassis and gene tools are limited, hampering the progress of developing engineering *Bifidobacterium.* Currently, most engineering efforts of *Bifidobacterium* are centred on *B. longum* and *B. breve,* with a few exceptions of other suitable microbial chassis. Additionally, various constitutive and inducible promoters were identified that were directly isolated from the *Bifidobacterial* genome. Interestingly, these gene promoters were functional in other microbial chassis such as *Bacillus* and *Escherichia coli.* The current inducible promoters used in *Bifidobacterial* engineering use sugar complexes as inducers to trigger genes regulated in the Bifid shunt pathway (Wang et al., 2019b).

While the current studies show promise of further developing *Bifidobacterium* as an engineering host, there is an increasing need for identifying more genetic tools that are better suited for detecting and responding to the various triggers in the host body. These efforts include identifying various regulatory elements from the pre-existing *Bifidobacterial* genome sequences and other closely related microbial species from the Actinobacterial taxa. Furthermore, the role of these engineered *Bifidobacteria* in the host-microbiome is scarcely studied and would require further investigation to better understand the impact and safety of using these microbes to treat diseases in the future.
