# Supplementary material for: Recent Development of Probiotic Bifidobacteria for Treating Human Diseases
Source: Front Bioeng Biotechnol. 2021 Dec 22;9:770248. doi: 10.3389/fbioe.2021.770248 (PMC8727868; doi:10.3389/fbioe.2021.770248)
Supplement: Supplementary file 1 [file Table1.doc]

Table S1. Current *Bifidobacterium* classification based on the NCBI taxonomy database (adapted from https://www.ncbi.nlm.nih.gov/Taxonomy/Browser/wwwtax.cgi).

| *Bifidobacterial* species | strains |
| --- | --- |
| *Bifidobacterium actinocoloniiforme* | *Bifidobacterium actinocoloniiforme* DSM 22766 |
| *Bifidobacterium adolescentis* | *Bifidobacterium adolescentis* ATCC 15703 *Bifidobacterium adolescentis* DSM 20087  *Bifidobacterium adolescentis* L2-32  *Bifidobacterium adolescentis* XVI-120 *Bifidobacterium adolescentis* XVI122 |
| *Bifidobacterium aemilianum* |  |
| *Bifidobacterium aerophilum* |  |
| *Bifidobacterium aesculapii* |  |
| *Bifidobacterium angulatum* | *Bifidobacterium angulatum* DSM 20098 = JCM 7096  *Bifidobacterium angulatum* F16_22 |
| *Bifidobacterium animalis* | *Bifidobacterium animalis* subsp. animalis  *Bifidobacterium animalis* subsp. lactis |
| *Bifidobacterium anseris* |  |
| *Bifidobacterium apousia* |  |
| *Bifidobacterium apri* |  |
| *Bifidobacterium aquikefiri* |  |
| *Bifidobacterium asteroides* | *Bifidobacterium asteroides* DSM 20089  *Bifidobacterium asteroides* PRL2011 |
| *Bifidobacterium avesanii* |  |
| *Bifidobacterium biavatii* | *Bifidobacterium biavatii* DSM 23969 |
| *Bifidobacterium bifidum* | *Bifidobacterium bifidum* ATCC 29521 = JCM 1255 = DSM 20456  *Bifidobacterium bifidum* BGN4  *Bifidobacterium bifidum* CECT 7366  *Bifidobacterium bifidum* DSM 20215  *Bifidobacterium bifidum* IPLA 20015  *Bifidobacterium bifidum* JCM 1254  *Bifidobacterium bifidum* LMG 13195  *Bifidobacterium bifidum* NCIMB 41171  *Bifidobacterium bifidum* PRL2010  *Bifidobacterium bifidum* S17  *Bifidobacterium bifidum* VIII-210 |
| *Bifidobacterium bohemicum* | *Bifidobacterium bohemicum* DSM 22767 |
| *Bifidobacterium bombi* | *Bifidobacterium bombi* DSM 19703 |
| *Bifidobacterium boum* | *Bifidobacterium boum* DSM 20432 |
| *Bifidobacterium breve* | *Bifidobacterium breve* 12L  *Bifidobacterium breve* 2L  *Bifidobacterium breve* 31L  *Bifidobacterium breve* 689b  *Bifidobacterium breve* ACS-071-V-Sch8b  *Bifidobacterium breve* CECT 7263  *Bifidobacterium breve* DPC 6330  *Bifidobacterium breve* DSM 20213 = JCM 1192  *Bifidobacterium breve* EX336960VC18  *Bifidobacterium breve* EX336960VC19  *Bifidobacterium breve* EX336960VC21  *Bifidobacterium breve* EX533959VC21  *Bifidobacterium breve* HPH0326  *Bifidobacterium breve* JCM 7017  *Bifidobacterium breve* JCM 7019  *Bifidobacterium breve* JCP7499  *Bifidobacterium breve* MCC 0121  *Bifidobacterium breve* MCC 0305  *Bifidobacterium breve* MCC 0476  *Bifidobacterium breve* MCC 1094  *Bifidobacterium breve* MCC 1114  *Bifidobacterium breve* MCC 1128  *Bifidobacterium breve* MCC 1340  *Bifidobacterium breve* MCC 1454  *Bifidobacterium breve* MCC 1604  *Bifidobacterium breve* MCC 1605  *Bifidobacterium breve* NCFB 2258  *Bifidobacterium breve* S27  *Bifidobacterium breve* UCC2003 |
| *Bifidobacterium callimiconis* |  |
| *Bifidobacterium callitrichidarum* |  |
| *Bifidobacterium callitrichos* | *Bifidobacterium callitrichos* DSM 23973 |
| *Bifidobacterium canis* |  |
| *Bifidobacterium castoris* |  |
| *Bifidobacterium catenulatum* | *Bifidobacterium catenulatum* DSM 16992 = JCM 1194 = LMG 11043  *Bifidobacterium catenulatum* subsp. kashiwanohense |
| *Bifidobacterium catulorum* |  |
| *Bifidobacterium cebidarum* |  |
| *Bifidobacterium choerinum* | *Bifidobacterium choerinum* DSM 20434 |
| *Bifidobacterium choladohabitans* |  |
| *Bifidobacterium choloepi* |  |
| *Bifidobacterium commune* |  |
| *Bifidobacterium coryneforme* | *Bifidobacterium coryneforme* DSM 20216 |
| *Bifidobacterium criceti* |  |
| *Bifidobacterium crudilactis* |  |
| *Bifidobacterium cuniculi* | *Bifidobacterium cuniculi* DSM 20435 |
| *Bifidobacterium dentium* | *Bifidobacterium dentium* ATCC 27678  *Bifidobacterium dentium* ATCC 27679  *Bifidobacterium dentium* Bd1  *Bifidobacterium dentium* JCM 1195 = DSM 20436  *Bifidobacterium dentium* JCVIHMP022 |
| *Bifidobacterium dolichotidis* |  |
| *Bifidobacterium erythrocebi* |  |
| *Bifidobacterium eulemuris* |  |
| *Bifidobacterium faecale* |  |
| *Bifidobacterium felsineum* |  |
| *Bifidobacterium gallicum* | *Bifidobacterium gallicum* DSM 20093 = LMG 11596 |
| *Bifidobacterium goeldii* |  |
| *Bifidobacterium hapali* |  |
| *Bifidobacterium imperatoris* |  |
| *Bifidobacterium indicum* | *Bifidobacterium indicum* LMG 11587 = DSM 20214 |
| *Bifidobacterium italicum* |  |
| *Bifidobacterium jacchi* |  |
| *Bifidobacterium lemurum* |  |
| *Bifidobacterium leontopitheci* |  |
| *Bifidobacterium longum* | *Bifidobacterium longum* 3_1_37DFAAB  *Bifidobacterium longum* AGR2137  *Bifidobacterium longum* BORI  *Bifidobacterium longum* D2957  *Bifidobacterium longum* DJO10A  *Bifidobacterium longum* E18  *Bifidobacterium longum* NCC2705  *Bifidobacterium longum* subsp. infantis  *Bifidobacterium longum* subsp. longum  *Bifidobacterium longum* subsp. suillum  *Bifidobacterium longum* subsp. suis  *Bifidobacterium longum* X-95 |
| *Bifidobacterium magnum* | *Bifidobacterium magnum* DSM 20222 |
| *Bifidobacterium margollesii* |  |
| *Bifidobacterium merycicum* | *Bifidobacterium merycicum* DSM 6492 |
| *Bifidobacterium minimum* | *Bifidobacterium minimum* DSM 20102 |
| *Bifidobacterium mongoliense* | *Bifidobacterium mongoliense* DSM 21395 |
| *Bifidobacterium moraviense* |  |
| *Bifidobacterium moukalabense* | *Bifidobacterium moukalabense* DSM 27321 |
| *Bifidobacterium myosotis* |  |
| *Bifidobacterium oedipodis* |  |
| *Bifidobacterium olomucense* |  |
| *Bifidobacterium panos* |  |
| *Bifidobacterium parmae* |  |
| *Bifidobacterium platyrrhinorum* |  |
| *Bifidobacterium polysaccharolyticum* |  |
| *Bifidobacterium porcinum* | *Bifidobacterium porcinum* DSM 17755 |
| *Bifidobacterium primatium* |  |
| *Bifidobacterium pseudocatenulatum* | *Bifidobacterium pseudocatenulatum* D2CA  *Bifidobacterium pseudocatenulatum* DSM 20438 = JCM 1200 = LMG 10505  *Bifidobacterium pseudocatenulatum* IPLA36007 |
| *Bifidobacterium pseudolongum* | *Bifidobacterium pseudolongum* AGR2145  *Bifidobacterium pseudolongum* PV8-2  *Bifidobacterium pseudolongum* subsp. globosum  *Bifidobacterium pseudolongum* subsp. pseudolongum |
| *Bifidobacterium psychraerophilum* | *Bifidobacterium psychraerophilum* DSM 22366 |
| *Bifidobacterium pullorum* | *Bifidobacterium pullorum* ATCC 49618  *Bifidobacterium pullorum* subsp. gallinarum  *Bifidobacterium pullorum* subsp. pullorum  *Bifidobacterium pullorum* subsp. saeculare |
| *Bifidobacterium ramosum* |  |
| *Bifidobacterium reuteri* | *Bifidobacterium reuteri* DSM 23975 |
| *Bifidobacterium rousetti* |  |
| *Bifidobacterium ruminantium* | *Bifidobacterium ruminantium* DSM 6489 |
| *Bifidobacterium saguini* | *Bifidobacterium saguini* DSM 23967 |
| *Bifidobacterium saimiriisciurei* |  |
| *Bifidobacterium samirii* |  |
| *Bifidobacterium scaligerum* |  |
| *Bifidobacterium scardovii* | *Bifidobacterium scardovii* JCM 12489 = DSM 13734 |
| *Bifidobacterium simiae* |  |
| *Bifidobacterium simiarum* |  |
| *Bifidobacterium stellenboschense* |  |
| *Bifidobacterium subtile* | *Bifidobacterium subtile* DSM 20096 |
| *Bifidobacterium thermacidophilum* | *Bifidobacterium thermacidophilum* subsp. thermacidophilum |
| *Bifidobacterium thermophilum* | *Bifidobacterium thermophilum* DSM 20210  *Bifidobacterium thermophilum* DSM 20212  *Bifidobacterium thermophilum* RBL67 |
| *Bifidobacterium tibiigranuli* |  |
| *Bifidobacterium tissieri* |  |
| *Bifidobacterium tsurumiense* | *Bifidobacterium tsurumiense* DSM 17777 |
| *Bifidobacterium vansinderenii* |  |
| *Bifidobacterium vespertilionis* |  |
| *Bifidobacterium xylocopae* |  |
| *unclassified Bifidobacterium* |  |
